# Correlation of DNA methylation of *DNMT3A* and *TET2* with oral squamous cell carcinoma

**DOI:** 10.1007/s12672-024-00866-9

**Published:** 2024-01-22

**Authors:** Xueming Li, Zaikun Li, Qingxi Gao, Yanan Peng, Yang Yu, Tenglong Hu, Wei Wang

**Affiliations:** 1 Department of Oral and Maxillofacial Surgery, The First Affiliated Hospital of Harbin Medical University, Harbin, 150001 China; 2 Key Laboratory of Hepatosplenic Surgery, Ministry of Education, The First Affiliated Hospital of Harbin Medical University, Harbin, China

## Abstract

Oral squamous cell carcinoma (OSCC) is the sixth most common malignancy worldwide. Abnormal epigenetic modifications, including DNA methylation, are hallmarks of cancer and implicated in the development of various tumors. DNA methylation is catalyzed by the DNA methyltransferase and ten-eleven translocation dioxygenase families, with *DNMT3A* and *TET2* being the most widely studied members, respectively. The correlation of methylation β values and clinical features was conducted in patients with OSCC in The Cancer Genome Atlas database. DNA methylation and protein expression levels of *DNMT3A* and *TET2* in tissues were analyzed with methylation-specific polymerase chain reaction (MSP) and western blotting. To evaluate the effects of *DNMT3A* and *TET2* on the biological characteristics of OSCC, cell proliferation was assessed with 5-ethynyl-2'-deoxyuridine, and cell migration capacity was quantified with wound healing and transwell assays. A survival analysis was performed with the Kaplan–Meier approach. The correlation between different methylation β values and clinical features was revealed. MSP revealed varying methylation degrees of *DNMT3A* and *TET2* in OSCC tissues. Furthermore, western blotting showed that the protein expression levels were significantly different in cancer and surrounding healthy tissue samples. In vitro experiments demonstrated that *DNMT3A* knockdown and *TET2* overexpression could inhibit the proliferation and migration of OSCC. Survival analysis revealed that patients with high *DNMT3A* methylation levels showed higher survival rates.

## Introduction

Cancer is a global health burden because of the lack of prevention and treatment options. Oral squamous cell carcinoma (OSCC) is a common malignancy, with high rates of metastasis, recurrence, and resistance to conventional chemotherapy. Every year, nearly 400,000 new cases and 200,000 deaths [1] are attributed to OSCC, showing a poor prognosis for most affected patients.

Abnormal epigenetic modification is a hallmark of cancer. Epigenetic modification factors, particularly DNA methylation, affect tumor occurrence, development, invasion, metastasis, and treatment resistance [2]. By changing the functional state of the regulatory regions without altering the DNA sequence, DNA methylation can significantly affect the gene expression. For example, hypermethylation of the gene promoter regions may lead to transcriptional silencing of certain tumor suppressors, while loss of methylation in the promoter regions may lead to decreased genomic stability and potentially activate viral sequences in the genome [3]. DNA methylation patterns are established through a coordinated mechanism catalyzed by various enzymes, including the DNA methyltransferase (DNMT) and ten-eleven translocation (TET) dioxygenase families, with *DNMT3A* and *TET2* being the most extensively studied members, respectively. DNMT3A is an enzyme responsible for the de novo methylation of cytosine residues and a modification frequently associated with gene silencing. TET2 is a demethylase enzyme that initiates a series of cytosine demethylation reactions, which perform antagonistic enzymatic activity.

DNA methylation is a crucial regulatory mechanism in the development and maintenance of acute myeloid leukemia, and *DNMT3A* mutations are implicated in various hematological tumors. Furthermore, *DNMT3A* is associated with chemical carcinogenicity and can lead to double-stranded DNA damage [4]. It is also involved in the amino acid metabolism. Methionine dependence is a hallmark of cancer. *DNMT3A* is a key gene in the methionine metabolism and predicts a poor cancer prognosis [5]. In addition, it is closely related to biological processes [6–9] and signaling pathways [10–12].

The demethylase *TET2* is a regulator of normal hematopoiesis, particularly bone marrow cell production [13–15]. In addition to frequent mutations in various hematological tumors, *TET2* is also associated with immune and inflammatory responses. *TET2* plays a vital role in various inflammatory conditions by regulating the immune response [16] and expressions of signaling networks and effectors during the initiation and regression of inflammation [17–19]. Simultaneously, *TET2* mutations occur in human solid tumors, and a reduced TET2 protein expression is prevalent in several types of cancers [20–25].

Although the pathogenic roles of *DNMT3A* and *TET2* in various tumors have been well-documented, their role in OSCC has rarely been studied. Some studies have shown their expressions in OSCC tissues [26–30]; however, their effects on the proliferation or migration of OSCC remain unclear. The aim of the present study was to determine whether or not *DNMT3A* and *TET2* could serve as latent biomarkers for the diagnosis and prognosis of OSCC.

## Material and methods

### Patients and methylation analysis

Clinical information and methylation chip data of 399 patients with OSCC was obtained from The Cancer Genome Atlas (TCGA) database (https://portal.gdc.cancer.gov/). The methylation β values of *DNMT3A* and *TET2* genes under different clinical characteristics were extracted. The Kolmogorov–Smirnov test were used to assess the mean methylation differences of all probes in each sample under different clinical settings and the methylation differences of each probe in different regions.

### Cell line and culture

OSCC cell line Cal27 was obtained from Procell and maintained in Dulbecco’s Modified Eagle Medium (DMEM; Gibco) supplemented with 10% fetal bovine serum (Gibco) and 1% penicillin_streptomycin at 37 °C under 5% CO2. The cells were subcultured using 0.25% trypsin at 80–90% confluency, and the medium was changed every 2 days.

### Tumor and surrounding healthy tissues

29 primary tumor and surrounding healthy tissue samples were obtained from the First Affiliated Hospital of Harbin Medical University. Qualified pathologists certified all tissues and determined the OSCC diagnosis. Informed consent was obtained from all patients. The study protocol was approved by the institutional ethics committee of the hospital. The study procedures were performed in accordance with the tenets of the Declaration of Helsinki. After collection, all the tissues were immediately preserved in liquid nitrogen for future use.

### Primer design

Primer3 was used to design primers for reverse-transcription polymerase chain reaction (RT-PCR). For the methylation-specific polymerase chain reaction (MSP), we used MethPrimer to design primer sequences.

### DNA extraction, bisulfite conversion, and MSP

The QIAamp DNA Mini Kit (Qiagen) was utilized to obtain genomic DNA from 29 fresh primary tumor tissues. The EZ DNA Methylation-Gold Kit (Zymo Research) was used for bisulfite conversion to detect the methylation status. In MSP, every 20 μL of the reaction mixture contained 2 μL of DNA, and amplification was performed using ABI 7500 (Thermo Fisher Scientific). PCR involved an initial denaturation step at 95 °C for 10 min, followed by 40 cycles at 95 °C for 15 s and 60 °C for 60 s.

### Western blotting

Six pairs of fresh primary tumor and surrounding healthy tissue samples stored in liquid nitrogen were used to extract total protein. Each pair of tissues was cut into 0.03 g pieces, washed with phosphate-buffered saline, and lysed with the radioimmunoprecipitation assay (RIPA) lysis buffer. Finally, the TissueMaster Handheld Homogenizer (Beyotime) was utilized to grind the tissues thoroughly. Proteins in the tissues were separated using 8% sodium dodecyl-sulfate polyacrylamide gel electrophoresis and transferred onto a 0.22 μm nitrocellulose membrane (Pall Corporation). The membrane was incubated overnight with the following primary antibodies (Abcam) after blocking in a 5% skim milk solution for 2 h: DNMT3A (rabbit polyclonal, 1:1000) and TET2 (rabbit polyclonal, 1:1000). β-actin antibody (1:1000) was utilized as the control.

### Transfection and expression validation

Small interfering *DNMT3A* (si-*DNMT3A*) was purchased from Hanheng Biotechnology, and the human *TET2* overexpression plasmid was purchased from Genechem. Transfection was conducted using the Lipofectamine 2000 (Invitrogen) or jetPRIME (PolyPlus transfection) transfection reagent. For RNA extraction, the AxyPrep Multisource Total RNA Miniprep Kit (Axygen) was used. For protein extraction, the RIPA lysis buffer (Beyotime) was used. Moreover, RT-PCR and western blotting were performed to confirm the knockdown and overexpression. β-actin antibody (1:1000) and GAPDH were utilized as the control.

### Cell proliferation assay

To evaluate cell viability, the Cal27 cell lines were seeded in a 96-well plate and cultured for 24 h. Cell proliferation was assessed with the BeyoClick^™^ EdU Cell Proliferation Kit (Beyotime), which labels cells in the S phase of the cell cycle. The number of EdU-positive cells was observed under an inverted microscope (Nikon) to determine the extent of cell proliferation.

### Wound healing assay

The Cal27 cell lines were seeded in a six-well plate until the formation of monolayer cells. A 200 μL tip was then utilized to produce a scratch, and the detached cells were washed away. Serum-free DMEM was added to the wells for cell culture. After incubating for 24 and 48 h, the scratches were photographed using an upright microscope (Leica).

### Transwell assay

To assess the migration capacity of Cal27, we used 6 mm transwell chambers with 8-μm pores (Corning). A serum-free medium was added to the chambers, while a regular medium was added to a 24-well plate in which the chambers had been placed. After 48 h, the cells on the lower side of the membrane were fixed with 4% paraformaldehyde and stained with crystal violet. The stained cells were then photographed using an upright microscope.

### Statistical analysis

Statistical analyses were performed using GraphPad Prism 9 software. Fisher’s exact test, Student’s *t*-test, Kaplan–Meier (KM) approach and one- and two-way analyses of variance were used, as appropriate. Statistical significance was set at a *p*-value < 0.05. Each experiment was conducted at least thrice.

## Results

### Methylation β values analysis results of *DNMT3A* and *TET2* genes under different clinical characteristics

**Fig. 1:**
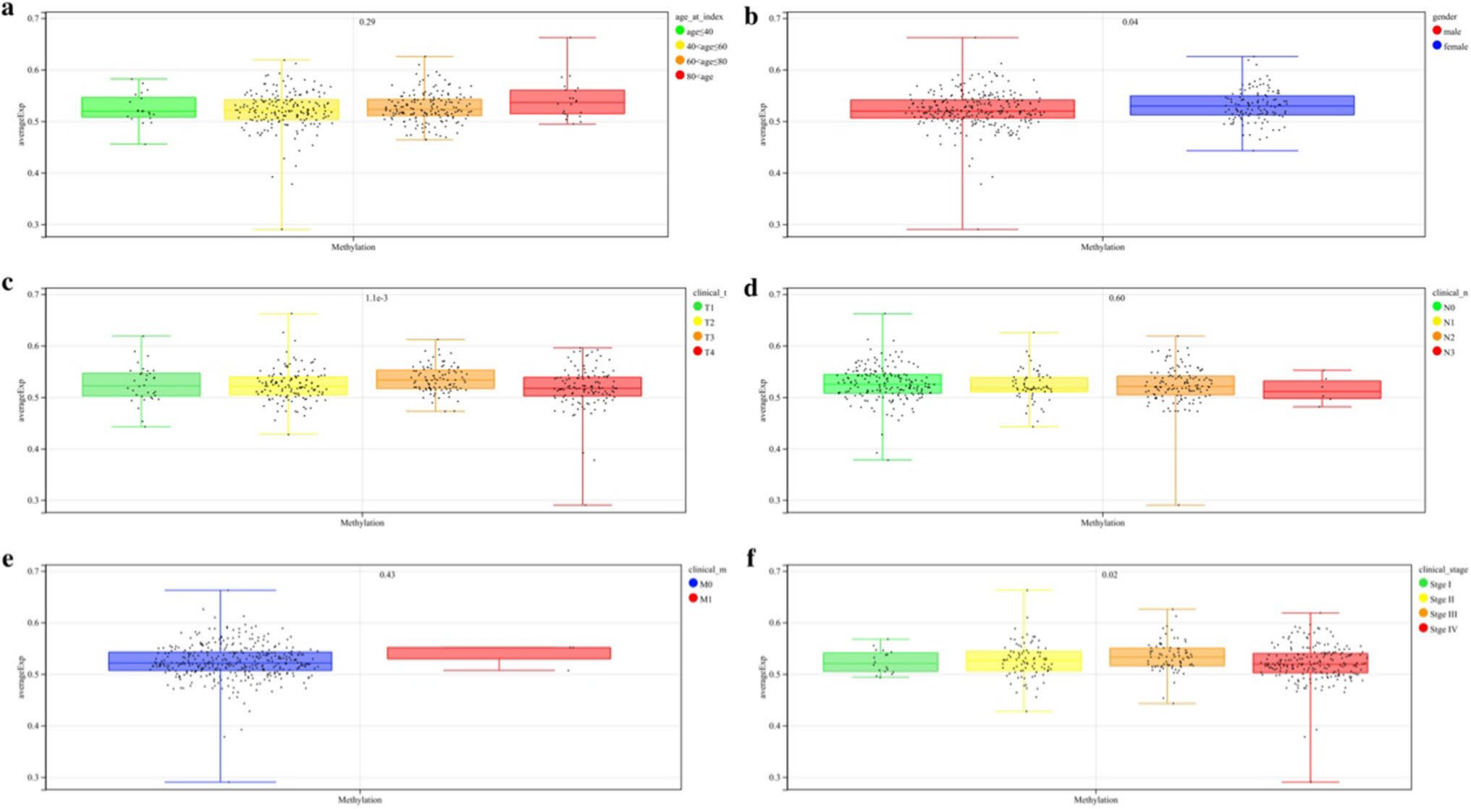
The correlation between the mean β value of all probes of *DNMT3A* and clinical features **a**: Age **b**: Gender **c**–**e**: TNM stage **f**: Clinical stage

**Fig. 2:**
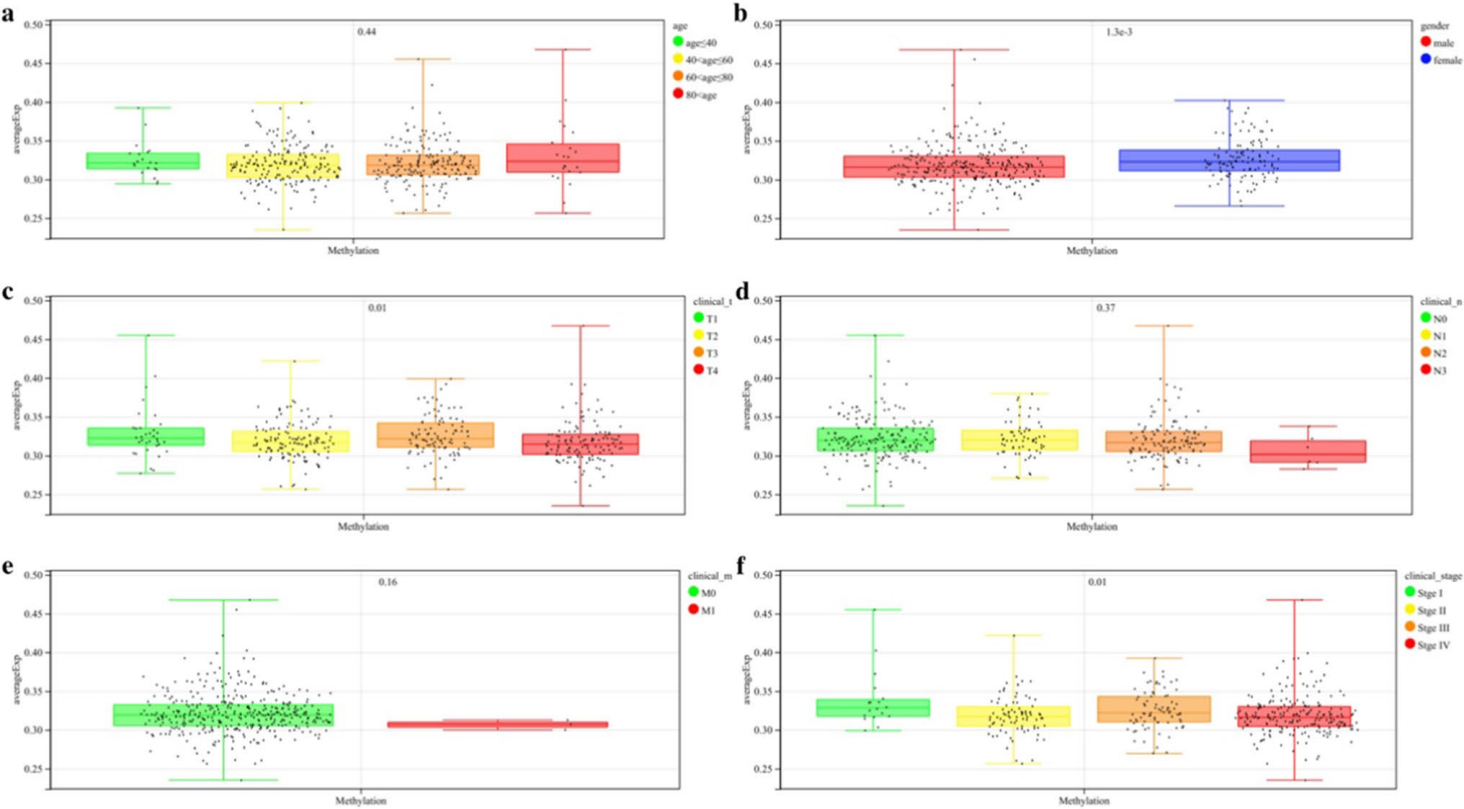
The correlation between the mean β value of all probes of *TET2* and Clinical features **a**: Age **b**: Gender **c**–**e**: TNM stage **f**: Clinical stage

**Fig. 3:**
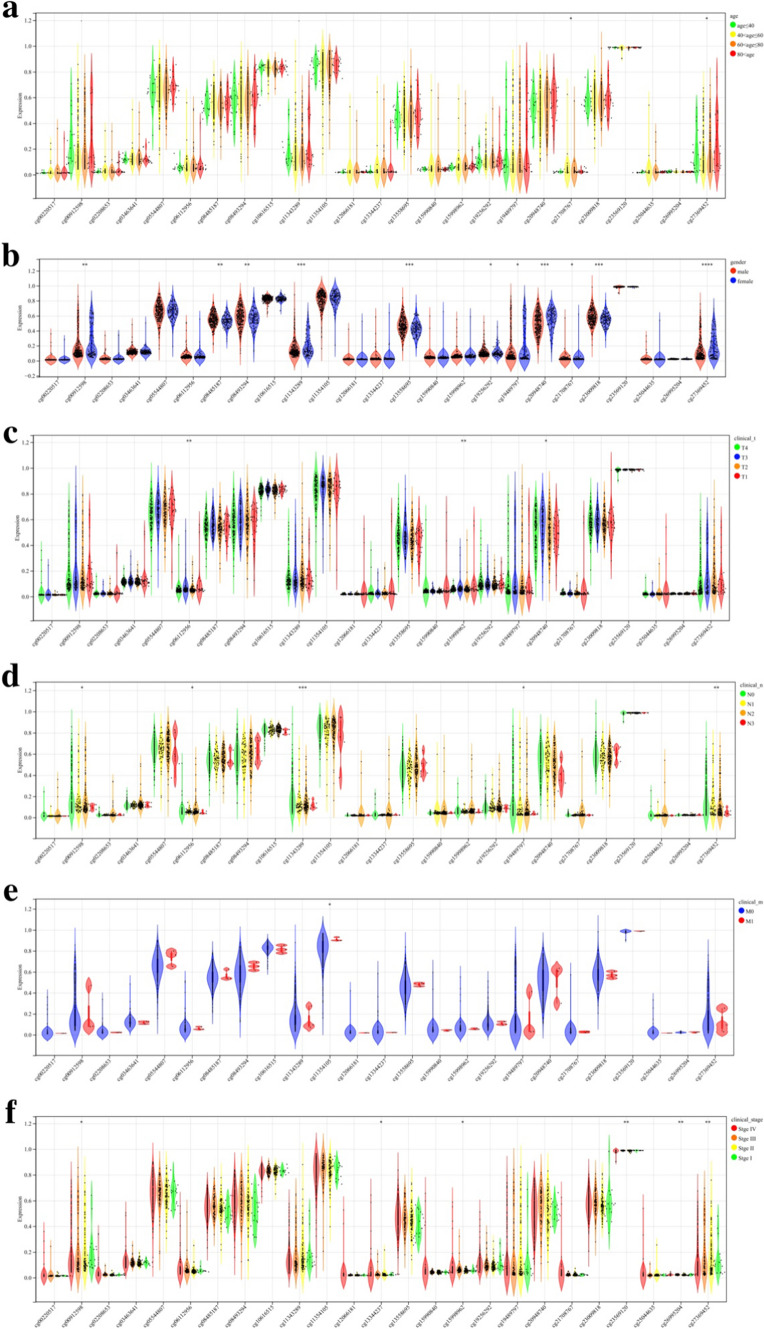
The correlation between the methylation β values of probes of *DNMT3A* in the CpG island region and clinical features **a**: Age **b**: Gender **c**–**e**: TNM stage **f**: Clinical stage

**Fig. 4:**
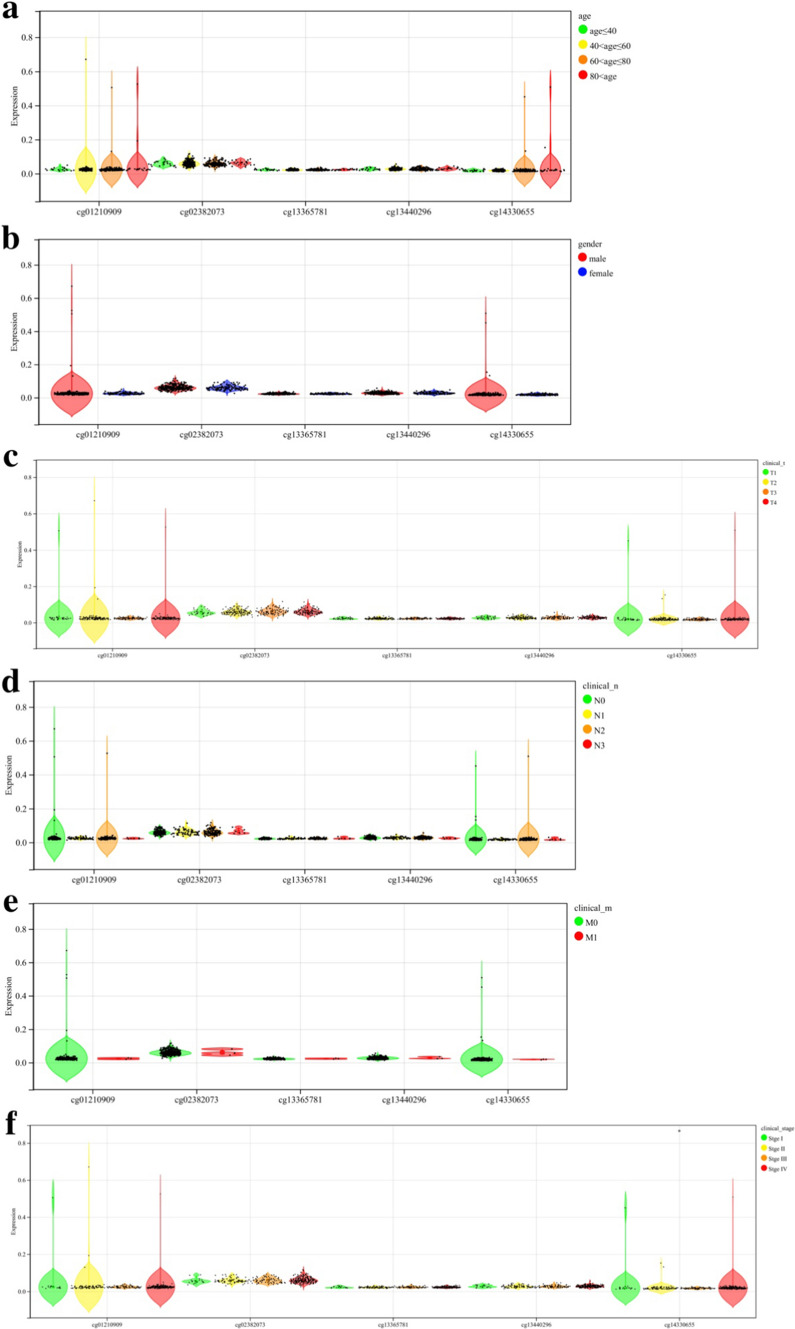
The correlation between the methylation β values of probes of *TET2* in the CpG island region and clinical features **a**: Age **b**: Gender **c**–**e**: TNM stage **f**: Clinical stage

We obtained methylation chip data and clinical information of 399 OSCC samples from the TCGA database. The correlation between the mean β value of all probes and age, gender, TNM stage, and clinical stage was analyzed. Methylation β values of *DNMT3A* and *TET2* were significantly correlated with gender, tumor size, extent of invasion, and clinical stage (*P* < 0.05; Figs. 1 and 2). We further grouped different CpG regions to examine the methylation β values of probes in different clinical groups. *DNMT3A* covers 25 probes in the CpG island region, seven probes in the downstream island region, 19 probes in the upper island region, and 15 probes in the Open_Sea region. *TET2* covers five probes in the CpG island region, three probes in the downstream island region, three probes in the upper island region, and four probes in the Open_Sea region. Six *DNMT3A* probes (cg00912598, cg13344237, cg15998962, cg23569120, cg26995204, cg27369452) and one *TET2* probe (cg14330655) in the CpG island region had a strong correlation with the clinical stage (*P* < 0.05; Figs. 3 and 4).

### Correlation of the methylation degrees of *DNMT3A* and *TET2* with the clinicopathological characteristics

The methylation degrees of *DNMT3A* and *TET2* were statistically analyzed in correlation with clinical and pathological features (gender, age, lesion site, tumor size, clinical stage, pathological stage, and lymphatic metastasis). The methylation degrees of *DNMT3A* and *TET2* differed significantly by pathological stage (Table 1, *P* < 0.05), but were not correlated with other clinicopathological parameters (*P* > 0.05). The methylation levels of the two genes changed with the degree of tumor differentiation; therefore, different protein expression levels were displayed at different stages.

**Table 1:** Relationship between clinicopathologic features and methylation degrees of *DNMT3A* and *TET2*

Pathological features	DNMT3A PM	DNMT3A FM	*P*	TET2 M	TET2 U	*P*
Total number of samples	21	8		13	16	
Gender			0.0667			0.6882
Man	17	3		8	12	
Woman	4	5		5	4	
Age			0.6968			> 0.9999
≤ 60	10	3		6	7	
> 60	11	5		7	9	
Tumor size			0.3715			0.4543
≤ 3 cm	8	1		3	6	
> 3 cm	13	7		10	10	
Location of lesions			0.6828			0.4515
Tongue and floor of the mouth	13	4		9	8	
Other	8	4		4	8	
Clinical staging			0.6706			0.7021
I-II	9	2		4	7	
III-IV	12	6		9	9	
Pathologic staging			0.0265*			< 0.0001****
High differentiation and medium differentiation	11	8		3	16	
Low differentiation	10	0		10	0	
Lymphatic metastases			0.3715			0.2256
Yes	8	1		6	3	
Not	13	7		7	13	

*PM* partially methylated, *FM* fully methylated, *M* methylated, *U* unmethylated

### Differences in DNMT3A and TET2 protein expressions in OSCC tissues

**Fig. 5:**
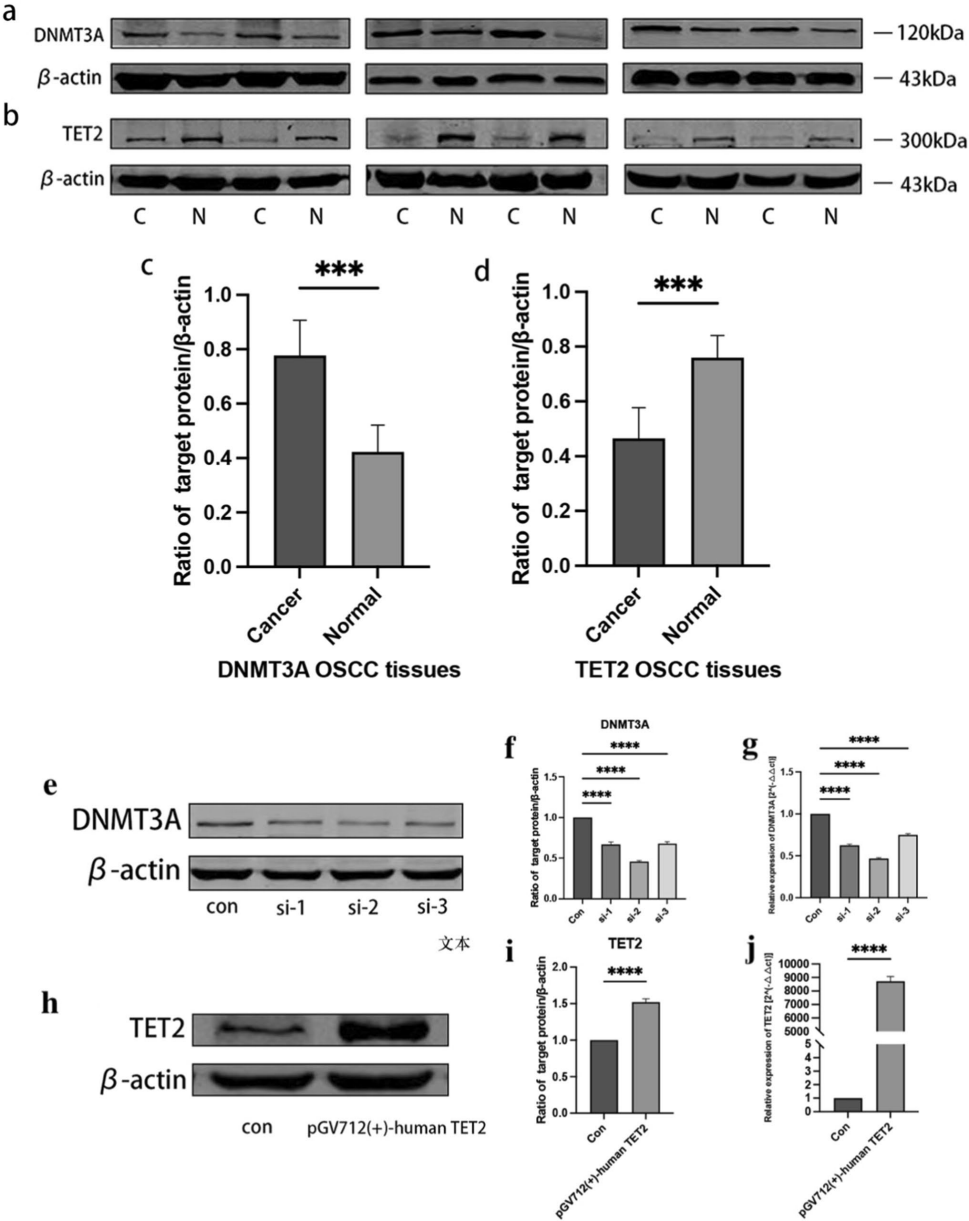
*DNMT3A* and *TET2* protein expressions in six pairs of OSCC samples and transfection effects of *DNMT3A* and *TET2* **a**, **b**: *DNMT3A* and *TET2* protein expression levels in tumor and surrounding healthy tissue samples **c**, **d**: Statistical analysis results of protein expressions **e**, **h**: *DNMT3A* and *TET2* protein expressions **f**, **i**: Statistical analysis results of protein expressions **g**, **j**: *DNMT3A* and *TET2* messenger RNA expressions

To assess the DNMT3A and TET2 protein expressions in OSCC, we performed western blotting of six pairs of primary tumor and surrounding healthy tissue samples. The DNMT3A expression was significantly higher in OSCC samples than in surrounding healthy tissue samples (*P* < 0.05, Fig. 5a), while the TET2 expression was the opposite (*P* < 0.05, Fig. 5b). Based on these results, we purchased si-*DNMT3A* and *TET2* overexpression plasmids for subsequent cell function experiments.

### Expression validations of *DNMT3A* and *TET2*

To determine the transfection effects of si-*DNMT3A* and *TET2* overexpression plasmids, we performed western blotting (*P* < 0.05, Fig. 5e, h) and RT-PCR (*P* < 0.05, Fig. 5g, j) to confirm the knockdown and overexpression, respectively. Both knockdown and overexpression showed good results.

### Effects of *DNMT3A* and *TET2* on OSCC cell proliferation

**Fig. 6:**
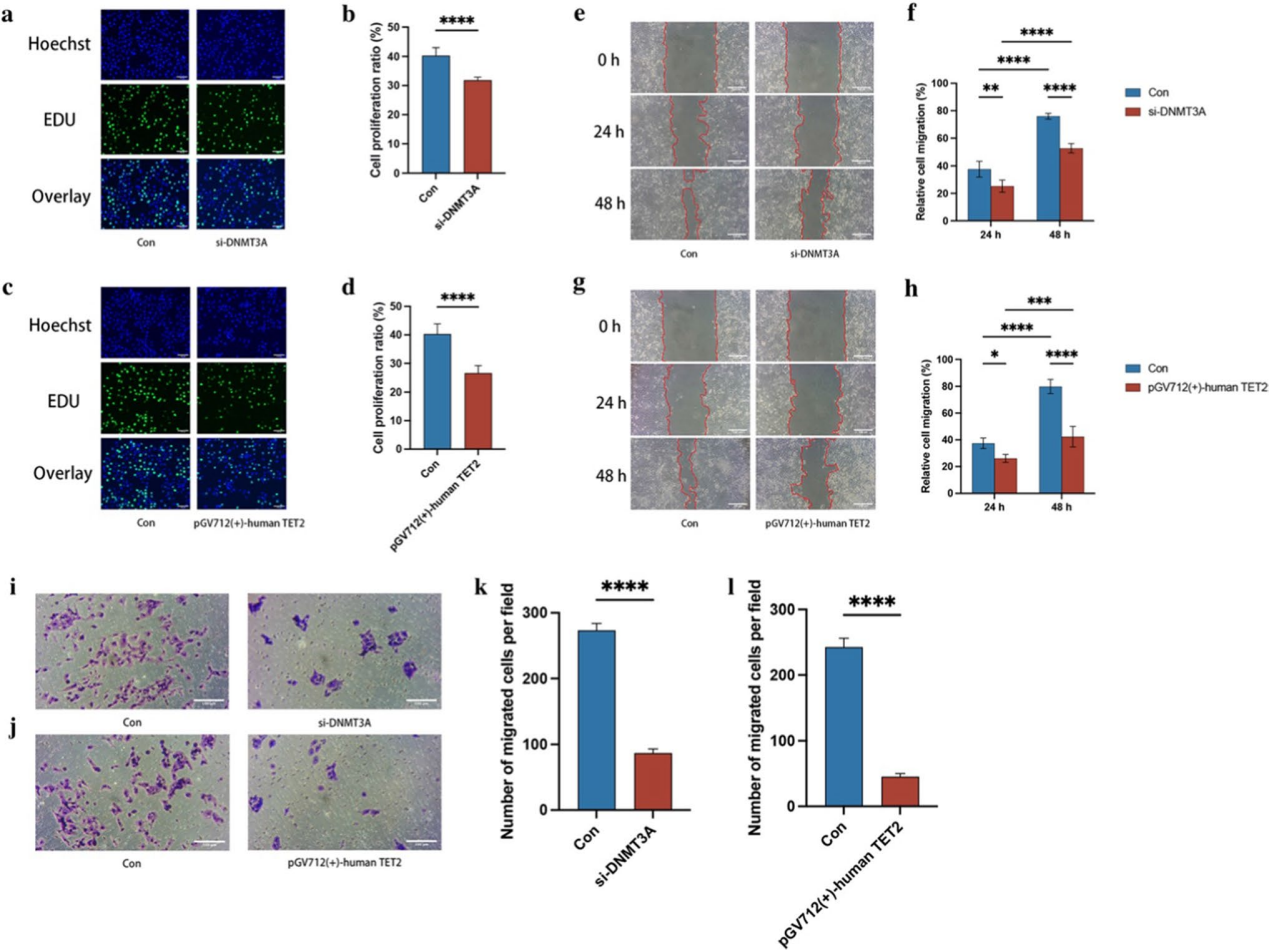
Proliferation and migration of OSCC cells **a**: Cells in the proliferation phase in the *DNMT3A* experimental group **c**: Cells in the proliferation phase in the *TET2* experimental group **b**, **d**: Statistical analysis results of proliferation **e**: Cal27 cell migration in the *DNMT3A* experimental group **g**: Cal27 cell migration in the *TET2* experimental group **f**, **h**: Statistical analysis results of migration **i**: Cal27 cell migration in the *DNMT3A* experimental group j: Cal27 cell migration in the *TET2* experimental group **k**, **l**: Statistical analysis results of migration (scale bar = 100 μm)

In the EdU cell proliferation experiment, the *TET2* overexpression group showed a significantly lower number of proliferating cells compared to the control group (*P* < 0.05; Fig. 6c). However, unlike the *TET2* overexpression experimental group, the *DNMT3A* knockdown experimental group showed no significant differences (*P* < 0.05; Fig. 6a). The number of proliferating cells was displayed by the amount of green fluorescence. The results suggested that *TET2* played a greater role in the proliferation of OSCC cells compared to *DNMT3A*.

### Effects of *DNMT3A* and *TET2* on OSCC cell migration

We conducted wound healing and transwell assays to assess the migratory ability of OSCC cells. The wound healing assay showed wound healing at 24 and 48 h. The *DNMT3A*-knockdown (*P* < 0.05, Fig. 6e) and *TET2* overexpression experimental groups (*P* < 0.05, Fig. 6g) showed significantly low wound healing rates. Similarly, in the transwell assay, compared to the control group, both experimental groups showed a reduced number of OSCC cells passing through the transwell to varying degrees (*P* < 0.05; Fig. 6i, j). These results suggested that *DNMT3A* and *TET2* played vital roles in cell migration inhibition. *TET2* displayed a weaker ability to deform OSCC cells, making it more difficult for cells to pass through pores. Compared to the *DNMT3A*-knockdown experimental group, the *TET2* overexpression experimental group showed a stronger ability to inhibit the migration of OSCC cells.

### Association between *DNMT3A* and *TET2* methylation levels and OSCC survival

**Fig. 7:**
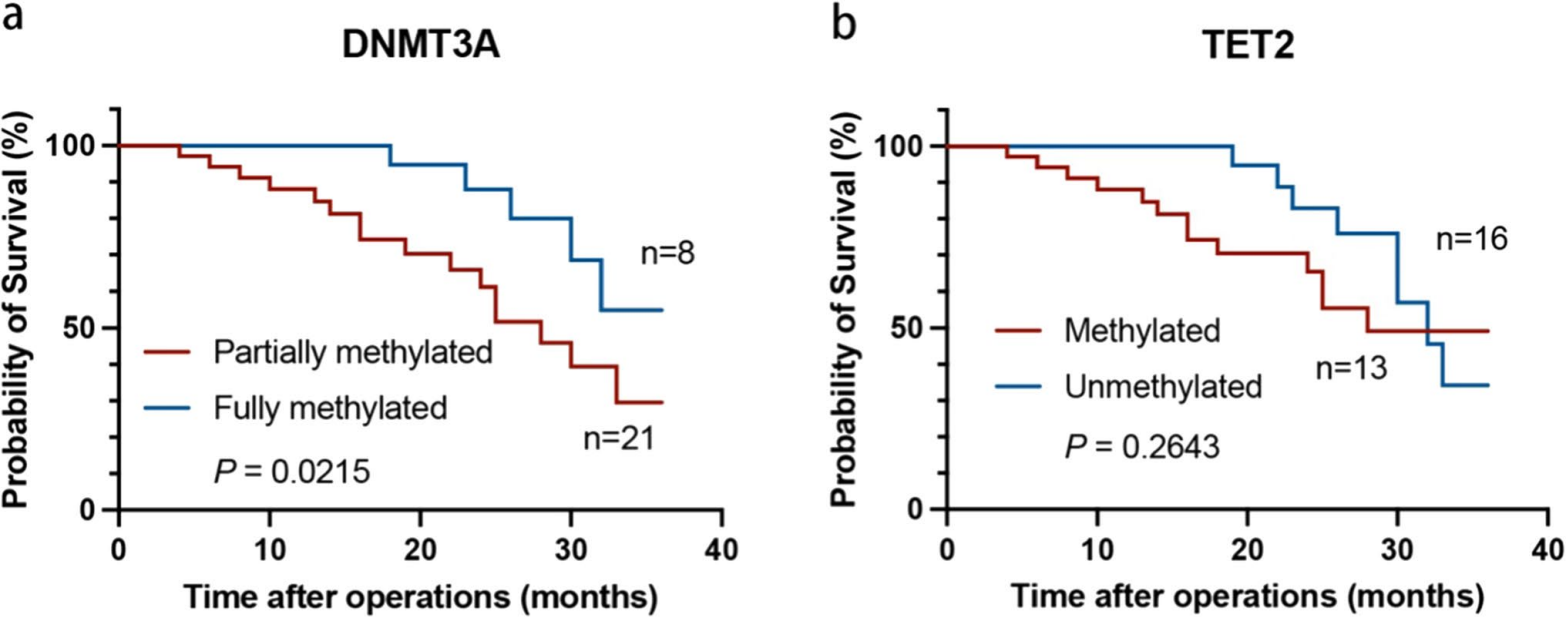
The association between *DNMT3A* and *TET2* methylation levels and OSCC survival **a**: The relationship of methylation levels of *DNMT3A* with OSCC survival **b**: The relationship of methylation levels of *TET2* with OSCC survival

According to the KM method, cases of high DNMT3A methylation levels showed higher survival rates (*P* < 0.05; Fig. 7a). However, TET2 methylation levels were not significantly associated with OSCC survival (*P* > 0.05; Fig. 7b). This result may be owing to the low number of cases and short follow-up time.

## Discussion

OSCC is the sixth most common malignancy worldwide, and its occurrence and development result from the gradual accumulation of various factors, including epigenetic factors. DNA methylation modifications affect genetic mutations and occur before tumor formation [31]. In general, the epigenetic state of cells is precisely regulated to maintain an appropriate state of differentiation. However, this carefully balanced genomic programming is disturbed in cancer, resulting in uncontrolled cell proliferation, impaired differentiation, and resistance to apoptosis [32]. Therefore, the DNA methylation status of target genes is a potential therapeutic target in cancer treatment and biomarker in early detection and prognostication of cancer. Consequently, it should be explored for improving cancer treatment outcomes.

In this study, the methylation degree revealed that as the cancer became less differentiated, *DNMT3A* promoter methylation decreased gradually, indicating that its expression was no longer inhibited. The level of global methylation of cancer increased, thereby promoting the malignant transformation of tumors. *DNMT3A* is involved in the oncogenic process of various tumors by regulating the level of promoter methylation. Yu et al. revealed that miR-26a-5p targeted *DNMT3A* to reduce the degree of global methylation in non-small cell lung cancer and restore the SFRP1 expression, thereby regulating cell viability and the stem-like phenotype by regulating the Wnt/β-catenin pathway [33]. Lu et al. found *DNMT3A* promoted the Warburg effect and tumor malignant biological behavior by inhibiting the miR-603 expression. Therefore, it is a potential therapeutic target for ovarian cancer [34]. Pang et al. demonstrated that MYC-recruited DNMT3A induced promoter methylation, resulting in miR-200b silencing, thereby promoting epithelial mesenchymal transformation and mammary globular formation in triple-negative breast cancer cells [35].

In the present study, *TET2* functioned as a tumor suppressor gene (TSG) in OSCC. The methylation degree revealed that *TET2* was predominantly unmethylated in tissues with a lower degree of malignancy, indicating that the TET2 expression was not inhibited. Further, as the degree of differentiation decreased, the degree of promoter methylation increased, and the expression was inhibited. Promoter methylation of TSGs can be an early occurrence in the development of tumors. This epigenetic alteration may confer advantages to cell growth, in turn promoting malignant transformation. TSGs that undergo abnormal promoter hypermethylation can encode various protein products, including DNA repair factors, cell cycle inhibitors, and cell adhesion receptors [36]. Hitchins et al. reported that promoter hypermethylation of *MLH1* and epigenetic silencing were associated with various cancers characterized by defects in mismatch repair [37]. Casalino et al. showed that *CDKN2A* encoded the cell cycle inhibitor *p16INK4A*, and loss of the p16INK4A expression because of hypermethylation of its promoter was an early event in breast and lung cancers [38]. Markou et al. selected five TSGs, involved in cancer cell differentiation, proliferation, apoptosis, adhesion, and metastasis for methylation evaluation, which in combination with circulating tumor cells and matched plasma-free DNA provided effective prognostic information for patients with early lung cancer [39]. These examples confirm that abnormal methylation contributes to the development of cancer by affecting genes involved in key cellular processes and suggest that the methylation degree of the promoter of TSGs change continuously during tumor development, which is an effective mean for the early detection, disease surveillance, and evaluation of the therapeutic efficacy.

The present study showed a higher protein expression of DNMT3A in tumor samples than in surrounding healthy tissue samples. The TET2 expression in cancer tissues was significantly reduced, and its recovery could effectively inhibit the malignant biological behavior of OSCC. Altered expression levels of DNMTs, particularly elevated DNMT3A levels, are common in multiple cancer samples and cell lines [40]. TET proteins regulate the equilibrium between DNA methylation and demethylation by regulating the dynamic transformation among cytosine, 5-mC, and 5-hmC [41]. However, missense and truncated TET mutations have been reported in nearly all types of solid tumors [42], and their reduced protein expression and 5-hmC levels are markers of many cancer types, including colorectal cancer [43], glioblastoma [24], cervical cancer [44], and pancreatic cancer [45]. The aforementioned studies are consistent with our findings, showing that modulating the DNMT3A and TET2 expression levels could effectively change the biological behavior of OSCC.

This was the first study to show that *DNMT3A* and *TET2* methylation levels change with the degree of cancer differentiation and that *DNMT3A* knockdown or *TET2* overexpression could effectively inhibit the proliferation and migration of OSCC. Although the oncogenic mechanisms of *DNMT3A* and *TET2* should be further explored, they have a wide range of applications as potential therapeutic targets for cancer.

## Data Availability

Data will be made available on request.
